# Esthetic Crown Lengthening Associated With Modified Lip Repositioning Surgery (Modified LipStat) in the Treatment of Excessive Gingival Display: A 6-Year Follow-Up Case Report

**DOI:** 10.1155/2024/3456235

**Published:** 2024-09-05

**Authors:** Cesar Augusto Signori Arruda, Luciano Melo Pratto, Estevo D'Agostini Derech, Guenther Schuldt Filho

**Affiliations:** ^1^ Department of Postgraduate Studies in Dentistry São Leopoldo Mandic University, Campinas, São Paulo, Brazil; ^2^ Department of Periodontology Nova Southeastern University, Fort Lauderdale, Florida, USA

**Keywords:** altered passive eruption, gingival aesthetics, gummy smile, lip repositioning surgery

## Abstract

Smiling expresses emotions and affects interpersonal relationships, influencing self-esteem and social life. Nowadays, concerns about aesthetics and access to the Internet have made people more informed and demanding about treatments for gingival smile. Excessive gingival display (EGD) is caused by a variety of factors that can act alone or in combination. These factors may include altered passive eruption (APE), vertical maxillary excess, a short upper lip, and hyperactive upper lip, among other factors that cause gingival hyperplasia. In this case report, the EGD was caused by vertical maxillary excess, hypermobility of the upper lip, and APE. The proposed treatment included two procedures: the Modified Lip Repositioning Surgery (Modified LipStat) Technique, with internal sutures to attempt to restrict the traction of the upper lip elevator muscles, and Esthetic Crown Lengthening (ECL) from the right first premolar to the left first premolar for the treatment of APE. The result provided an aesthetic improvement of the smile, with harmonization in relation to the size of the teeth. The mobility of the upper lip in spontaneous smiles remained reduced up to 6 months of follow-up; however, there was a partial relapse in the position of the upper lip during spontaneous smiling at the end of 6 years of follow-up.

## 1. Introduction

The smile plays a significant role in expression and appearance. The demand for aesthetics increases each year, and achieving the “perfect smile” has become an essential goal for many patients. Therefore, in addition to the teeth, the condition of the oral tissues, gingival contour, and lip position are factors that affect the final aesthetic of a smile. Creating the ideal smile is a challenge, as the treatment requires meticulous planning and a multidisciplinary approach [1].

Excessive gingival display (EGD), commonly described as a gummy smile, presents an aesthetic problem and can lead to dissatisfaction with the smile [2]. A study compared oral health and quality of life between individuals with and without EGD. Fifty-three patients with EGD and another 53 control patients were selected. Through a questionnaire that evaluated numerous aspects, it was observed that the degree of aesthetic satisfaction in patients with EGD was 21.1%, while in control patients, it was 78.9% [3].

A smile is typically considered pleasant when the upper teeth are fully exposed. However, the degree of gingival exposure considered aesthetically pleasing varies according to cultural norms. Generally, a gingival display of no more than 2–3 mm is considered acceptable, whereas an exposure greater than 3 mm is usually regarded as unattractive [4].

There are many factors that cause EGD, and this condition often results from the interaction of multiple etiologies [5]. These include altered passive eruption (APE), vertical maxillary excess, a short upper lip, excessive mobility of the upper lip, and other conditions that cause gingival enlargement. Unfortunately, there is still considerable confusion among healthcare professionals regarding diagnosis and treatment. Appropriate treatment techniques should be selected based on the identification and definitive diagnosis of the causes of EGD [4]. Therefore, more than one procedure may be performed to achieve the desired aesthetic [4, 6].

The most well-known technique for correcting a gummy smile is Esthetic Crown Lengthening (ECL). However, when crown proportions are adequate, other treatment approaches should be considered. Lip Repositioning Surgery (LipStat) can be utilized when indicated. This is a minimally invasive procedure with few postoperative complications, aimed at shortening the depth of the vestibular sulcus to limit the retraction of the upper lip elevator muscles [4].

In this case report, two surgical techniques were employed: the Modified Lip Repositioning Surgery (Modified LipStat), with internal sutures, aimed at repositioning the upper lip and limiting its movement during smiling [7, 8], and ECL, in order to treat the APE, improve the height-to-width ratio of the dental crowns, and reduce the amount of exposed gingiva during smiling. The objective of this study is to present the results of this case after 6 years of follow-up, enabling the dentist to analyze the long-term effects of the two techniques used in the pursuit of an ideal smile.

## 2. Case Report

**Figure 1:**
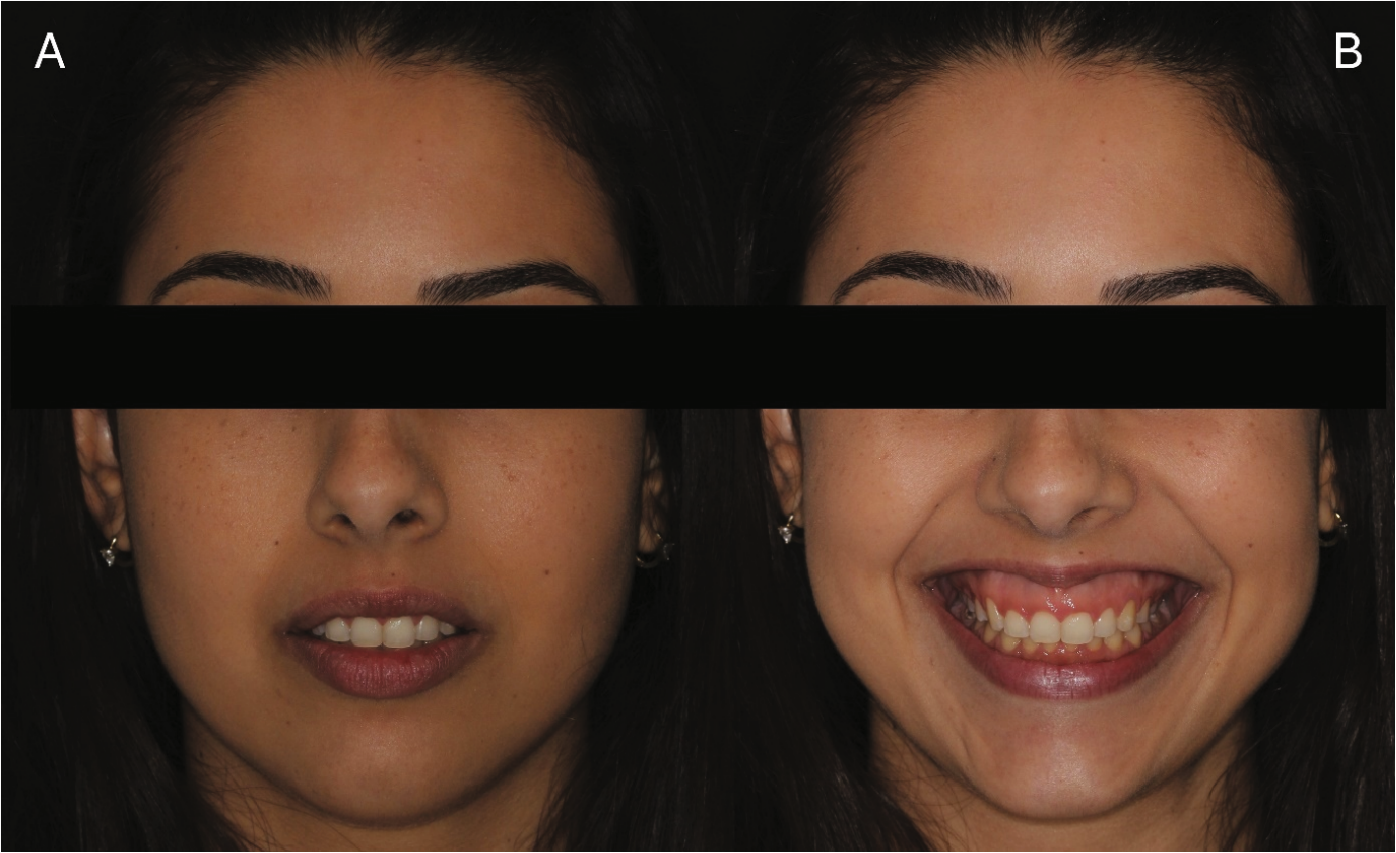
Frontal views of the patient. (A) Vertical excess of the maxilla with the upper lip at rest without natural veiling. (B) Smile with an EGD of 8 mm on average.

A 25-year-old female patient presented to the Brazilian Dental Association of Santa Catarina with the chief complaint of EGD (Figure 1(A)). The patient had Class II vertical maxillary excess, hyperactive upper lip, and disproportionate clinical crowns in the height/width ratio, caused by APE. She was unwilling to undergo orthognathic surgery.

An EGD was observed during the smile, extending from the right first upper premolar to the left first upper premolar. The height of gingival display during a spontaneous smile averaged 8 mm (Figure 1(B)). The upper lip height was 21 mm (measured from the base of the nose to the inferior border of the upper lip at rest), which is considered normal for women.

A treatment plan was proposed with the patient's informed consent, to first perform a Modified LipStat in the initial stage, and after 3 months, proceed with the second stage involving ECL.

The medications used for the first surgical stage were a corticosteroid (dexamethasone 30 mg), initiated 2 h before the procedure and continued for 2 days; an antibiotic (amoxicillin 500 mg), initiated 2 h before the procedure and continued for 7 days; and an analgesic (dipyrone 1 g) for pain management. After intraoral disinfection with 0.12% chlorhexidine and extraoral disinfection with 0.5% chlorhexidine, local anesthesia with 2% mepivacaine and 1:100,000 epinephrine was administered to the infraorbital nerve and the buccal surface region, bilaterally.

**Figure 2:**
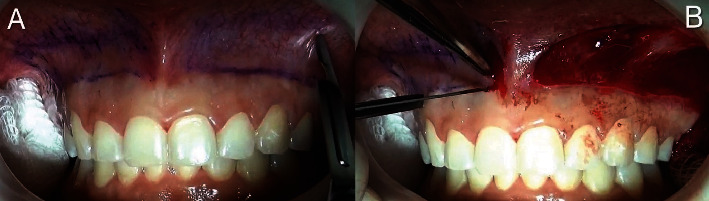
Intraoral view. (A) Markings for mucosal removal. (B) Removal of the band and exposure of the connective tissue on the left side.

The incision outline was initially marked with a sterile pencil (Figure 2(A)). The lower edge of the outline was slightly above the mucogingival junction, and the upper edge was delineated parallel to the lower edge, at a distance one and a half times greater than the height of the gingival display [4]. Specifically, the distance between the upper and lower margins of the outline averaged 12 mm. The two lines were connected at the mesial ends of the second premolars on each side to create a rectangular outline.

The incision was made using a No. 15C scalpel blade (Swann-Morton). A partial thickness dissection was performed at the mucogingival junction, and all the epithelium was excised along the rectangular outline, exposing the underlying connective tissue (Figure 2(B)).

**Figure 3:**
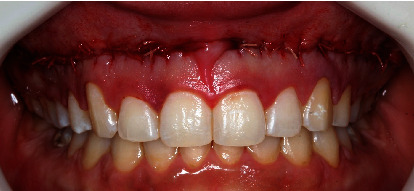
Intraoral view. Simple sutures with 5-0 Vicryl (Ethicon) providing closure of the upper and lower edges by first intention.

The entire strip of mucosa between the two incisions was removed. Deep internal horizontal mattress sutures were placed to limit muscle movement and approximate the surgical edges. Simple external sutures were used to unite the surgical edges. In both areas, 5-0 Vicryl absorbable sutures (Ethicon) were used (Figure 3). The labial frenulum was preserved to ensure the correct alignment of the lip midline with the dental midline. No myotomies were performed.

The patient was instructed to follow a diet of cold, soft foods for 2 days and to apply ice packs for 2 days (20-min sessions on each side of the face). The sutures were cleaned with cotton swabs soaked in 0.12% chlorhexidine for 7 days (after brushing). Other postoperative instructions were given, and the patient was discharged.

**Figure 4:**
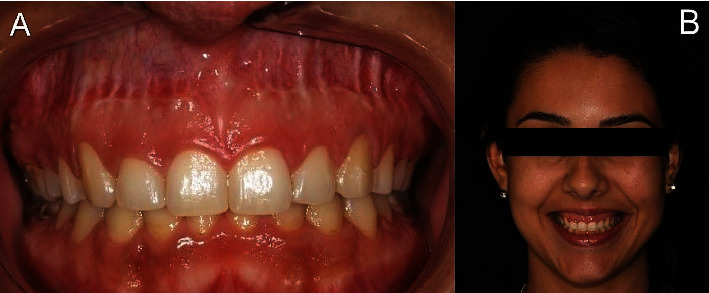
30-day postoperative period of Modified LipStat. (A) Visible scar coinciding with the mucogingival line. (B) EGD of 4.5 mm on average.

The patient was re-evaluated 7 days postoperatively. She reported minimal discomfort with a sensation of tightness at the site. Healing was considered good for this period. After 14 days, the patient was re-evaluated and reported no discomfort, and the external sutures were removed. At 30 days postoperatively, there was a visible scar line (Figure 4(A)), consistent with other reports in the literature. The gingival display decreased to an average of 4.5 mm (Figure 4(B)).

**Figure 5:**
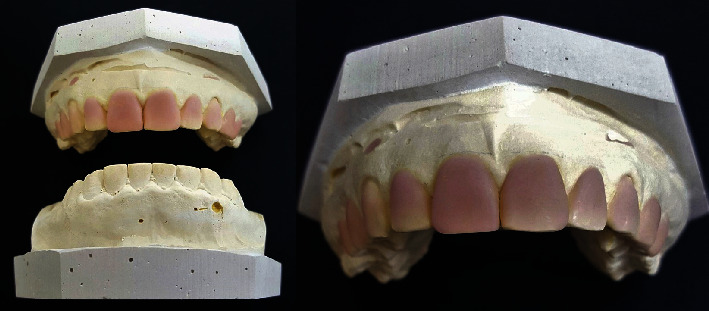
Gypsum model of diagnostic waxing. The new shape and height of the teeth is defined by waxing.

**Figure 6:**
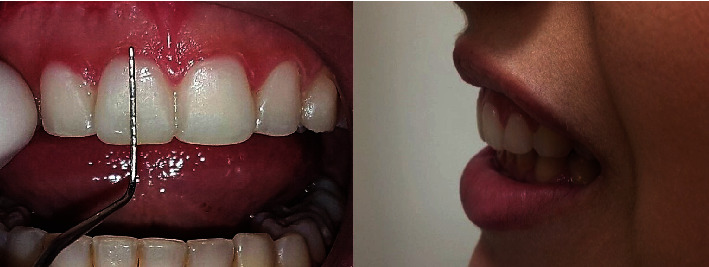
Temporary crowns installed for visual assessment of new tooth dimensions and adjustment of disocclusion guides. Intraoral and profile view of the mock-up for the patient to adapt and approve the treatment.

After a 3-month follow-up, the Aesthetic Crown Lengthening (ACL) surgery was planned. A cone beam computed tomography (CBCT) scan was performed to locate the cementoenamel junction of each tooth to be treated. Based on these measurements, a diagnostic wax-up was created (Figure 5), and a mock-up was made (Figure 6) for the patient's approval.

**Figure 7:**
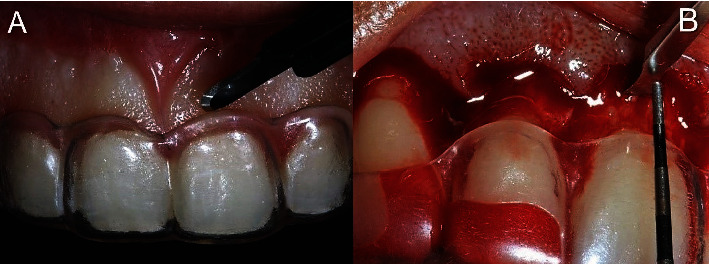
ECL. (A) Initial incision using the surgical guide as a reference to remove excess keratinized gingiva. (B) Osteoplasty 3 mm above the margin of the surgical guide, demarcated with a micro Ochsenbein chisel.

A surgical guide was fabricated based on the mock-up. The preoperative and anesthetic protocol was repeated. With the surgical guide in position, the excess keratinized gingiva was removed using a No. 15C scalpel blade (Swann-Morton) (Figure 7(A)). A full-thickness flap was elevated from the right first premolar to the left first premolar. Since the patient had a medium gingival biotype, osteoplasty was planned 3 mm above the cervical margin of the surgical guide. To delineate this margin, a micro Ochsenbein chisel (Quinelato) was used (Figure 7(B)).

**Figure 8:**
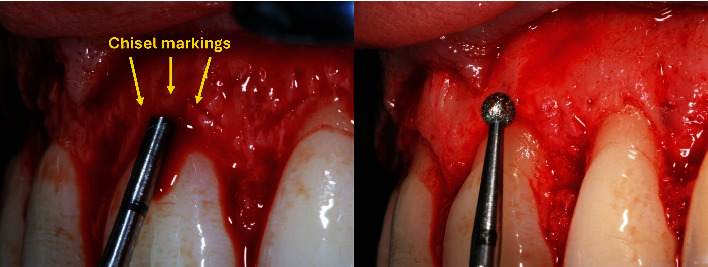
Osteoplasty 3 mm above the cementoenamel junction.

The osteoplasty was performed using a multifluted bur with cutting edges only on the apical section (Komet) to avoid cutting enamel or dentin. Osteoplasty was refined with a high-speed spherical diamond bur (Komet) according to the cementoenamel junction of all the teeth involved (Figure 8).

**Figure 9:**
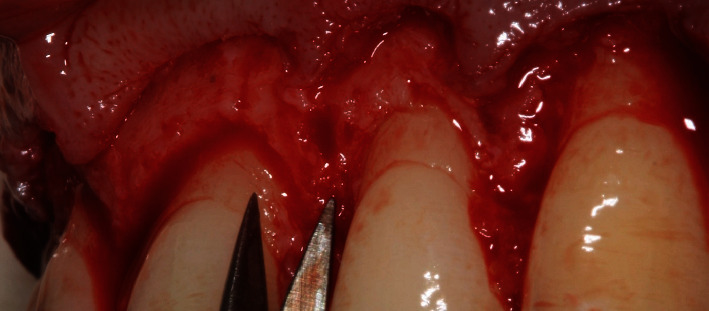
De-epithelialization of the papillae with curved microscissors (Schwertz) to remove the epithelium on the papillae for future coverage with the flap.

The papillae were de-epithelialized with curved microscissors (Schwertz) to overlap the vestibular flap (Figure 9).

**Figure 10:**
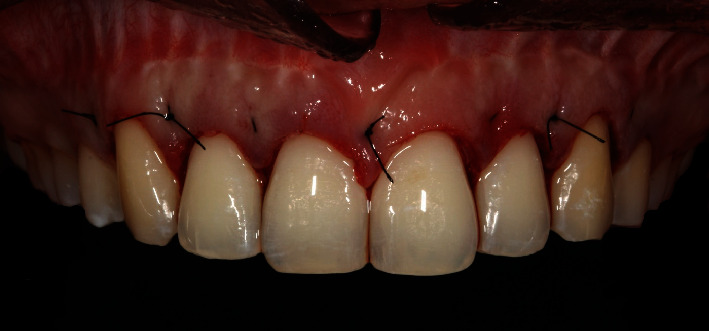
Intraoral view. Final stage of ACL surgery with flap repositioned using 5-0 Nylon sutures (Ethicon).

The flap was closed with suspensory sutures over the papillae using 5-0 Nylon sutures (Ethicon), involving the palatal surface of the dental elements (Figure 10). The same postoperative instructions from the first surgery were given to the patient.

**Figure 11:**
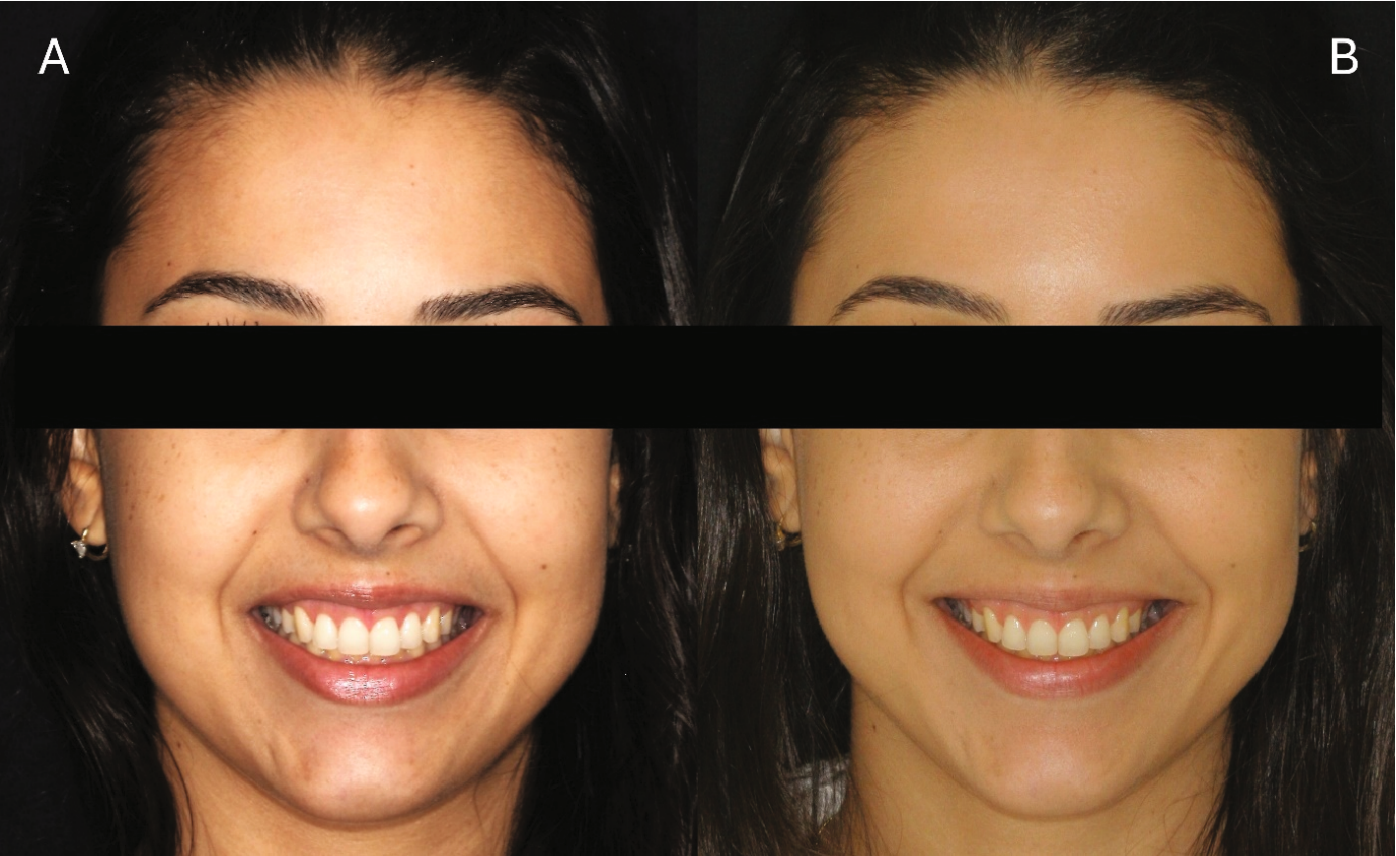
Front view. (A) Postoperative period of 4 months of Modified LipStat and 1 month of ECL. (B) Postoperative period of 6 months of Modified LipStat and 3 months of ECL.

After 7 days, the sutures were removed. The patient reported minimal discomfort and no edema. At the 30-day evaluation, proper healing of the gingival tissues was confirmed, and there was no need for esthetic restorations (Figure 11(A)). After 6 months of follow-up, the reduced gingival display was maintained (Figure 11(B)).

**Figure 12:**
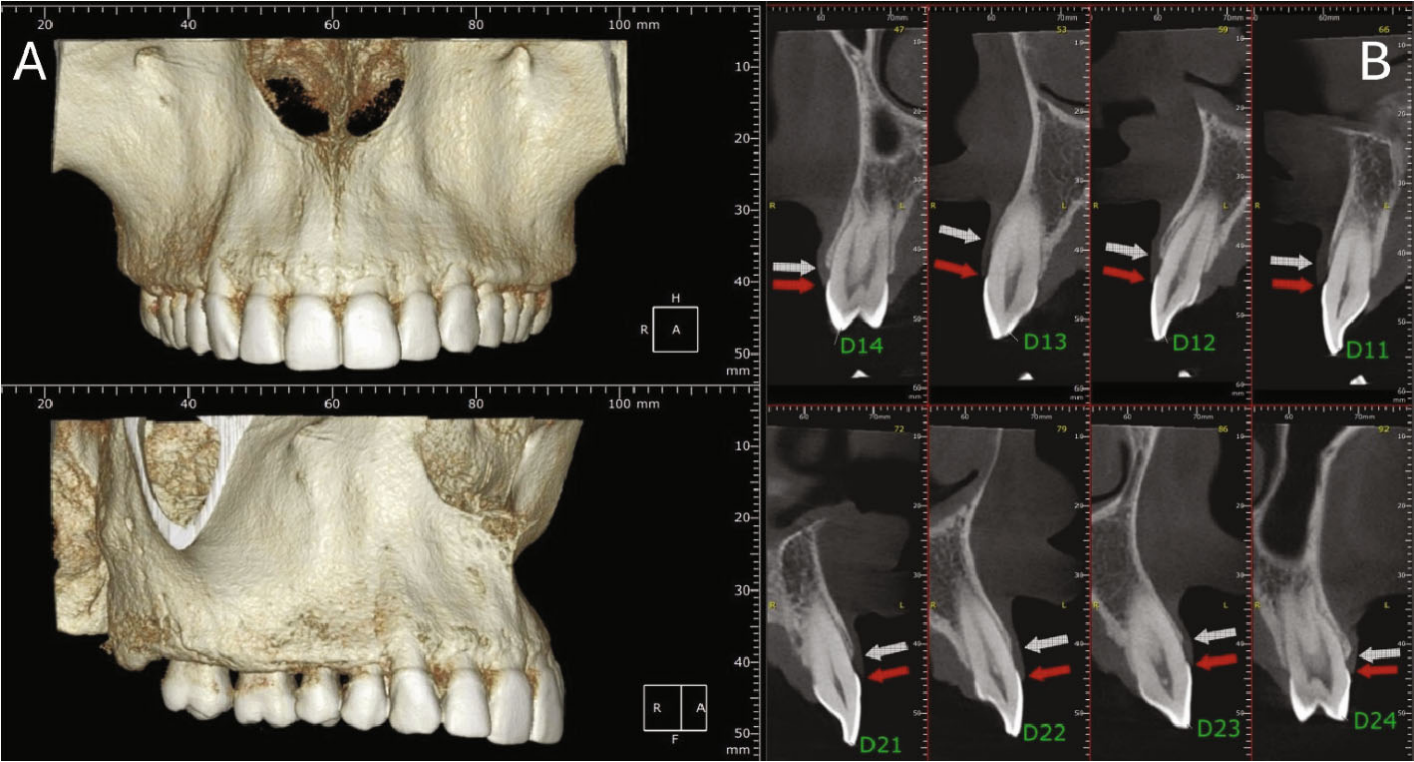
CBCT of the midface. (A) Alveolar bone remodeling on the buccal aspect. (B) New positions of the bone and gingival tissues in relation to the cementoenamel junction.

After 6 years of follow-up, a new CBCT was performed to analyze the new bone volume in the midface. Remodeled alveolar areas in the maxillary region are noted (Figure 12(A)). It is possible to confirm the new position of the bone crest in relation to the cementoenamel junction (Figure 12(B)).

**Figure 13:**
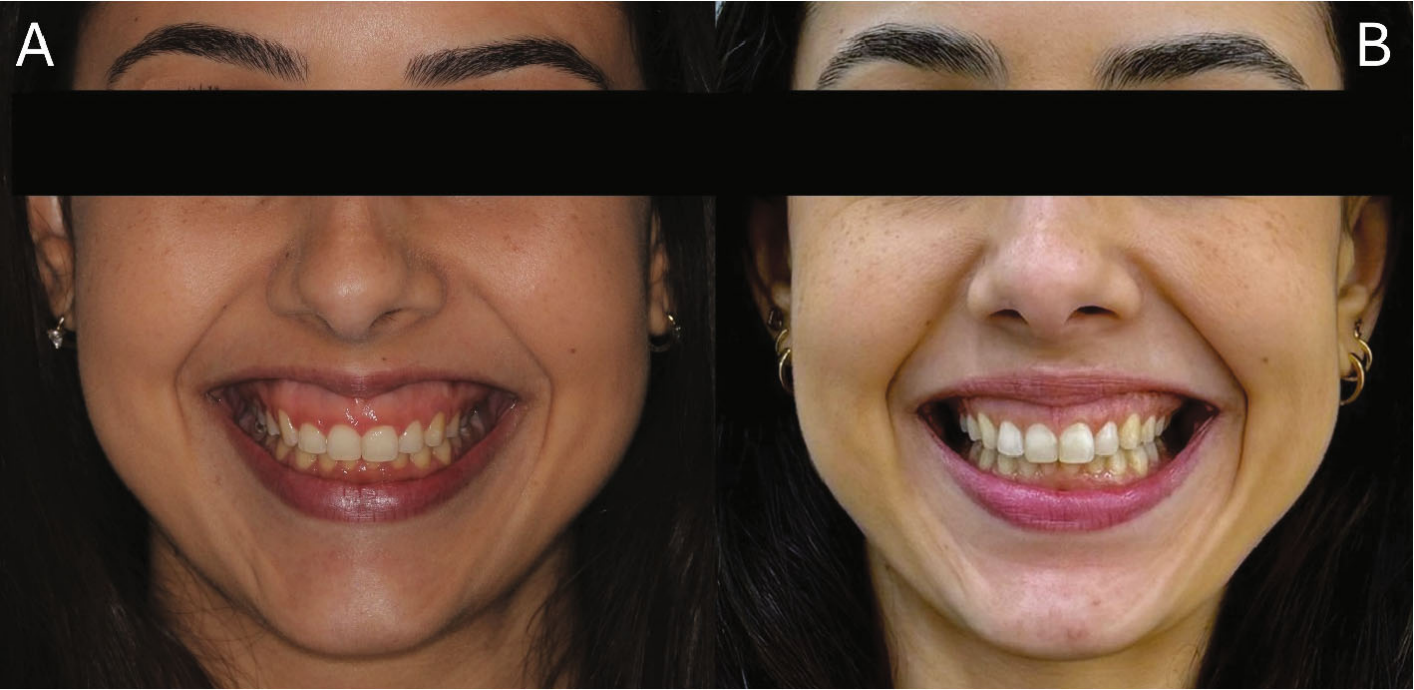
Front view. (A) Initial appearance of the spontaneous smile. (B) Appearance of the spontaneous smile after 6 years of follow-up.

Figure 13(A) illustrates the patient's initial condition during spontaneous smiling. Figure 13(B) shows the spontaneous smile 6 years postoperatively. After 6 years of follow-up, the proportion of dental crowns remained adequate following the ECL; however, there was partial relapse in the position of the upper lip during spontaneous smiling after the Modified LipStat procedure. Despite this, the patient was satisfied with the treatment outcome.

## 3. Discussion

For many years, dental aesthetic problems addressed by dentists were limited to the teeth, without considering the gums. However, it is now well established that these structures must be in balance to ensure an aesthetically pleasing appearance. Gingival exposure greater than 3 mm during smiling is considered EGD, also known as a gummy smile, which is viewed as unattractive in many cultures [9–12]. To achieve an ideal smile, it is essential for all dental, gingival, skeletal, and muscular structures to be in harmony [4, 9]. The present case showed an average exposure of 8 mm during spontaneous smiling, which is considered the threshold for indicating orthognathic surgery [4].

The literature classifies a gummy smile according to its different etiologies and mentions the possibility of a combination of these factors [4, 13–15]. The most recent classifications confirm that this variety of factors can act independently or in combination. These factors include APE, vertical maxillary excess, short upper lip, and hyperactive upper lip, among others that cause gingival enlargement [4]. In this case report, the patient presented with three causal factors for the gummy smile: vertical maxillary excess, hyperactivity of the upper lip (HUL), and APE.

Gingival recontouring is the most commonly used procedure in the treatment of EGD. The dentist can plan and perform the ECL procedure with greater precision using a 3D-printed guide, as it directs the removal of bone and soft tissues. The digital approach provides tools for achieving excellent treatment outcomes and facilitates communication between the dentist and the patient [16]. For the case in question, with significant gingival exposure and three etiologies involved, planning the ECL with a mock-up aided in visualizing the results prior to surgery. This made it possible to predict the new height of the teeth and predict the reduction of the exposed gingival band. The surgical guide ensured the desired proportion of the dental crowns as confirmed by the mock-up.

According to some reports in the literature, with the proper diagnosis and correct sequence of therapy, the Modified LipStat combined with ECL can be predictably used to treat EGD and enhance smile aesthetics [13, 17]. Recent research evidence indicates that APE and HUL are the two main soft tissue–based etiologies of EGD [18]. In this case, the Modified LipStat was performed before ECL. However, when these etiologies are associated with EGD, the authors consider that the best sequence is to start with the ECL procedure. This approach reduces the band of exposed gingiva during smiling, which should be measured again to plan the removal of the epithelial tissue band during LipStat.

Many cases of EGD have multiple etiologies and require more than one technique to achieve desirable aesthetic results [19–21]. Treatment should be tailored to each type of etiology, and it may be necessary to combine two or more techniques to resolve the issue [13, 16–18, 22–24]. With the partial recurrence in this case report after 6 years of follow-up, the possibility of orthognathic surgery or performing another Modified LipStat surgery remains as treatment options during continued follow-up.

The literature indicates that LipStat for the treatment of mild to moderate degrees of EGD is a less invasive procedure with fewer postoperative complications and faster recovery compared to orthognathic surgery [4, 14, 18, 25–27]. In the case in question, the patient reported greater discomfort in the postoperative period of the Modified LipStat compared to the postoperative period of ECL.

Contraindications for the Modified LipStat include cases with severe vertical maxillary excess (> 8 mm). These severe skeletal deformities should preferably be treated with orthognathic surgery [4, 14]. This is similar to the case report in question, which presented with an average exposure of 8 mm. A reduced height of attached gingiva may result in scar exposure during smiling. In these situations, the possibility of scar exposure at the mucogingival junction is a limitation of this technique and should be clearly explained to the patient [18]. In the case report, there was a high band of attached gingiva, but the patient was still warned about the risk of scar exposure during smiling in the postoperative period.

Reports in the literature show postoperative complications such as discomfort, bruising, edema of the upper lip, and occasionally mucocele formation due to the cutting of minor salivary glands [14]. In the case in question, precautions were taken during the Modified LipStat to prevent mucocele formation by carefully removing any exposed minor salivary glands after de-epithelialization and exposure of the underlying connective tissue. Rare complications include paresthesia and transient paralysis in cases of excessive removal of connective tissue [28]. Our team also took care to remove the minimum amount of connective tissue during the mucosa excision. More invasive approaches, such as muscle detachment, can increase the prevalence and duration of complications [3, 12, 14, 18, 22, 27].

Although some reports indicate a high prevalence of recurrence and suture loss, other studies show long-term stability. Previous reports indicate a higher chance of recurrence, especially in cases less suitable for this technique [20, 21, 29–31]. For this current case, during the Modified LipStat, we sought to perform sutures (internal and external) with good tension to limit the involved tissues and avoid suture loss until the removal of the external sutures on the 14th day.

Certain preoperative procedures should be performed, such as administering analgesics and cleaning the surgical site. When appropriate and necessary, prophylactic antibiotic therapy may also be indicated. Postoperatively, it is essential to manage pain and inflammation to ensure a comfortable recovery period, with specific instructions provided to the patient [27]. In our case report, in addition to the guidelines, the patient was preoperatively medicated with corticosteroids and antibiotics during both surgical stages to prevent edema and infection, respectively.

A systematic review conducted in 2018 investigated cases of EGE treated with LipStat. They observed an average reduction of 3.4 mm in gingival exposure, suggesting that the technique could be successfully used to treat EGE [1]. Corroborating the literature, this case report showed an average reduction of 3.5 mm in gingival exposure after the Modified LipStat, confirmed during the first months of follow-up.

Precise case selection is crucial to reduce the risk of recurrence in LipStat, and if followed correctly; the results will be sustainable for months [9, 32]. The stability of the lip in its new position is essential for the success of the procedure. Therefore, the use of appropriate suture techniques for a sufficient period is recommended to limit the action of the upper lip elevator muscles [8, 33–35]. For this current case, 12 internal horizontal mattress sutures and additional simple external sutures were performed, maintained for 14 days.

Regarding the Modified LipStat, a 2021 study with four patients demonstrated good results, with stability over an average follow-up of 3.5 years [7]. Another 2021 study evaluated a group treated with the conventional LipStat technique and another group treated with the Modified LipStat. In the group treated with the conventional technique, there was a slight increase in gingival exposure over 3 years. In the group treated with the Modified LipStat, higher satisfaction and more stable results were observed over 3.5 years of follow-up [36]. A 2023 study also compared long-term clinical results in two groups, one treated with the Modified LipStat and the other with the conventional LipStat. The results showed stability and no recurrence with the Modified LipStat up to 1 year of follow-up [34]. The satisfaction level of patients undergoing the Modified LipStat was significantly higher compared to those undergoing the conventional technique [37]. Another 2024 study compared results in two groups, with and without internal sutures. Both groups showed complete recurrence after 6 months of follow-up [35]. Based on several studies reported in recent years, it is important to emphasize that partial or total recurrence of LipStat can lead to dissatisfaction and compromise the dentist–patient relationship. Although the patient in the current case report showed satisfaction with the achieved result, the authors believe that, in addition to explaining the benefits, the inherent risks of this surgical lip repositioning technique should be well emphasized prior to treatment.

## 4. Conclusion

As this was a clinical case with significant initial gingival display (average of 8 mm), the patient was informed about the limitations of the results with the techniques used. After 6 years of follow-up, despite the partial relapse in the position of the upper lip during a spontaneous smile, the average gingival display remained reduced compared to the initial condition. Communicating all limitations to the patient before initiating treatment is crucial for maintaining a good relationship between the dentist and the patient.

According to the literature, ECL is demonstrated as a predictable treatment with favorable outcomes when performed according to appropriate standards. However, the Modified LipStat with internal horizontal mattress sutures, as well as the long-term evaluation of relapse, is an issue that needs to be extensively analyzed. The authors believe that more longitudinal studies with variations in internal sutures and longer maintenance periods for external sutures are necessary to determine the efficacy and stability of this treatment modality.
